# Zinc as a Drug for Wilson’s Disease, Non-Alcoholic Liver Disease and COVID-19-Related Liver Injury

**DOI:** 10.3390/molecules26216614

**Published:** 2021-10-31

**Authors:** Pierpaolo Coni, Giuseppina Pichiri, Joanna Izabela Lachowicz, Alberto Ravarino, Francesca Ledda, Daniela Fanni, Clara Gerosa, Monica Piras, Ferdinando Coghe, Yukio Gibo, Flaviana Cau, Massimo Castagnola, Peter Van Eyken, Luca Saba, Marco Piludu, Gavino Faa

**Affiliations:** 1 Department of Medical Sciences and Public Health, University of Cagliari, 09042 Monserrato, Italy; ppconi@tiscali.it (P.C.); albertoravarino@tiscali.it (A.R.); ledda_francesca@tiscali.it (F.L.); danielafanni@aoucagliari.it (D.F.); clarge@tiscali.it (C.G.); monipiras@hotmail.com (M.P.); flacau@tiscali.it (F.C.); gavinofaa@gmail.com (G.F.); 2 Dipartimento Servizi di Diagnosi e Cura, Azienda Ospedaliero-Universitaria di Cagliari (A.O.U.), University of Cagliari, 09024 Cagliari, Italy; coghe.f@tiscali.it; 3 Hepatology Clinic, 1-34-20 Muraimachiminami, Matsumoto, Nagano 399-0036, Japan; gibo@gibo-clinic.com; 4 Laboratorio di Proteomica e Metabonomica-Istituto di Ricovero e Cura a Carattere Scientifico Fondazione Santa Lucia, 00013 Rome, Italy; maxcastagnola@outlook.it; 5 Department of Pathology, Genk Regional Ziekenhuis, 3600 Genk, Belgium; petervaneyken@skynet.be; 6 Department of Radiology, Azienda Ospedaliero Universitaria (A.O.U.), di Cagliari—Polo di Monserrato s.s. 554, 09045 Monserrato, Italy; lucasabamd@gmail.com; 7 Department of Biomedical Sciences, University of Cagliari, 09042 Monserrato, Italy; mpiludu@unica.it; 8 UOC Anatomia Patologica, AOU Cagliari, University of Cagliari, 09124 Cagliari, Italy

**Keywords:** zinc, COVID-19, Wilson’s disease, non-alcoholic liver disease, drug therapy

## Abstract

Zinc is the second most abundant trace element in the human body, and it plays a fundamental role in human physiology, being an integral component of hundreds of enzymes and transcription factors. The discovery that zinc atoms may compete with copper for their absorption in the gastrointestinal tract let to introduce zinc in the therapy of Wilson’s disease, a congenital disorder of copper metabolism characterized by a systemic copper storage. Nowadays, zinc salts are considered one of the best therapeutic approach in patients affected by Wilson’s disease. On the basis of the similarities, at histological level, between Wilson’s disease and non-alcoholic liver disease, zinc has been successfully introduced in the therapy of non-alcoholic liver disease, with positive effects both on insulin resistance and oxidative stress. Recently, zinc deficiency has been indicated as a possible factor responsible for the susceptibility of elderly patients to undergo infection by SARS-CoV-2, the coronavirus responsible for the COVID-19 pandemic. Here, we present the data correlating zinc deficiency with the insurgence and progression of Covid-19 with low zinc levels associated with severe disease states. Finally, the relevance of zinc supplementation in aged people at risk for SARS-CoV-2 is underlined, with the aim that the zinc-based drug, classically used in the treatment of copper overload, might be recorded as one of the tools reducing the mortality of COVID-19, particularly in elderly people.

## 1. Introduction

Zinc (Zn) is the second most abundant trace element in the human body after iron. The zinc content in the adult body ranges from 1.4 to 2.3 g, with about 85% of the total amount localized in muscles and bones. According to recent findings, the brain is the organ with the highest Zn content, exceeding 10 times the zinc concentration in the liver and serum [1], but contrasting data can be found [2]. Such discrepancies come from the fact that the data are mostly based on zinc content from biopsies and sections of tissues, assuming that this reflects the total zinc content of the respective live tissue [3].

Zinc liver content is correlated with gestational age and decreases in the postnatal period [4]. Only 0.1% of total body zinc is found in plasma, where zinc atoms are bound to albumin [5], α-2-micro-globulin and transferrin [6]. Together with iron, copper, manganese, and selenium, zinc is a fundamental trace metal in human physiology, being an integral component of about 10% of the human proteome. Even if zinc has no redox potential, as other listed trace elements, it represents a key component in more than 300 enzymes and multiple transcription factors [7]. Studies counting the zinc proteins encoded in the human genome revealed that about 3000 human proteins are potential zinc-binders [8].

Zinc deficiency increases risk for infectious diseases (16% of all deep respiratory infections worldwide [9]), autoimmune disorders, and cancer [10,11,12]. Mild zinc deficiency is largely subclinical, and according to the World Health Organization’s (WHO) assumptions at least one third of the world population have zinc deficiency [9]. The highest incidence of patients affected by zinc deficiency is among those with chronic obstructive pulmonary disease (COPD), bronchial asthma, cardiovascular diseases, autoimmune diseases, kidney diseases, dialysis, obesity, diabetes, cancer, atherosclerosis, liver cirrhosis, immunosuppression, and liver damage [11,13]. Plasma zinc concentration corresponds only to 0.1% of the zinc in the body and widely depends on plasma zinc-transporters [14], thus plasma zinc concentration is not a reliable biomarker of cell/body zinc content. However, plasma zinc/albumin ratio could be considered as a surrogate marker for functional zinc status [14]. Nowadays, zinc deficiency is associated with low plasma concentration of zinc (<70 μg/dL), which are frequently detected during infections or in the elderly and in patients with chronic disease comorbidities that predispose to severe forms of COVID-19 [15].

Zinc is commonly used in the treatment of patients with documented zinc deficiency, which occurs in many types of liver disease, anorexia, anemia, growth retardation, abnormal immune function, abnormal nitrogen metabolism, hypogeusia, impaired reproductive capacity, coarse and sparse hair growth, flaking seborrhea of the skin, impaired connective tissue metabolism, behavioral defect, hypogonadism, and geophagia [16]. Recent studies put emphasis on zinc dyshomeostasis in neurological disorders [17] (e.g., brain injury, stroke, seizure, neurodegeneration [18]), α-1 antitrypsin polymorphism [19] and diabetes [20,21].

In this review, we present the role of zinc in biological processes and how its deficiency leads to different pathologies. In addition, we summarize the literature data of zinc-drug pharmacology and clinical trials for the use of zinc as a drug in prevention and treatment of numerous diseases. Finally, we discuss the beneficial effects of zinc therapy in Wilson’s disease (WD), non-alcoholic liver disease (NALD) and COVID-19-related liver injury.

## 2. Zinc Absorption and Metabolism

Zinc homeostasis is driven mainly by the gastrointestinal system, especially the small intestine, liver and pancreas, where absorption of exogenous zinc and gastrointestinal secretion and excretion of endogenous zinc occurs [22]. Zinc absorption depends on proper dietary intake, and is greatly affected by its intestinal availability [2]. Under regular physiologic conditions, zinc uptake does not saturate; although, the quantification of absorbed zinc is influenced by the zinc secretion into the gut. Numerous studies in human models with the use of stable zinc isotopes delivered important data on zinc absorption [23]. For instance, Zn in aqueous solutions is absorbed quite efficiently (at a rate of 60–70%), while absorption from solid dietary products is less effective, and greatly influenced by zinc content and presence of zinc competitors, e.g., other metal ions (iron and calcium), and zinc-chelating molecules [10]. Of note, zinc status conditionate zinc absorption, and zinc-deficient humans absorb zinc more efficiently, whereas humans with high-zinc diet have reduced zinc absorption [10]. Populations that followed habitual vegetarian diets have dietary zinc intakes and serum zinc concentrations significantly lower compared with non-vegetarians [10].

**Scheme 1:**
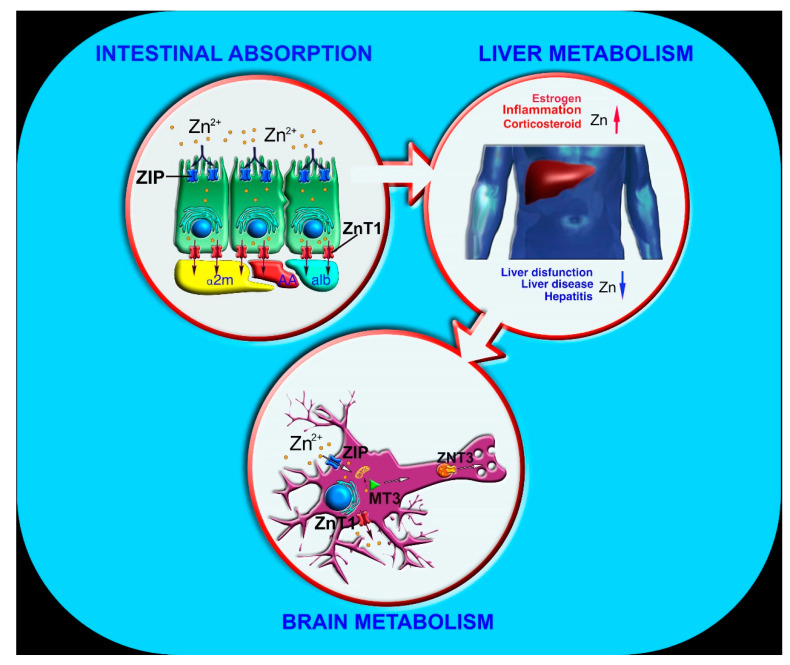
Zinc metabolism is finely regulated in the body in order to limits its potential toxic effect. The zinc dietary intake is regulated in the intestine through several zinc transporters proteins (ZnT) and by iron-regulated transporter proteins (ZIP). Zinc bound to α2 -macroglobulin (a2m), albumin (alb) and other proteins and amino acid (AA) is distributed in the body. Zinc is prevalently stored in skeletal muscle, bone, liver and brain. Liver plays a central role in zinc metabolism and, in general hepatitis, liver disfunction or liver diseases are associated with low levels of this trace element. In the brain, zinc is necessary for the regular central nervous system functions.

During the digestion process, Zn is released from food as free ions, which are absorbed in the duodenum [24] and jejunum [25] (Scheme 1). Zinc absorption, transfer, and excretion are accomplished by two large classes of transporters: Zinc transporters (ZnT) and zinc-regulated and iron-regulated transporter proteins (Zip). These proteins have opposite roles within the cell. The Zip family move zinc from the extracellular space into the cells, increasing cytoplasmic zinc concentrations, while ZnT is mostly decreasing intracellular zinc. Zinc is uptaken at the intestinal brush border membrane, where it is transported from the lumen into the enterocytes by the Zrt-, Irt-like protein (ZIP)4 (solute carrier (SLC39A4) transporters [26,27] and ZnT-1 (SLC30A1)), which is a basolateral membrane protein exporting zinc on the basolateral side of enterocytes into the portal blood [28]. In addition, the basolaterally localized transporters ZIP5 (SLC39A5) and ZIP14 (SLC39A14) transfer Zn from the blood circulation into enterocytes [29,30]. Moreover, ZnT-5 variant B (SLC30A5B) at the apical membrane of enterocytes [31,32] transports both luminal Zn into enterocytes and cellular ions back into the lumen [32,33]. Recently, research data showed that polymorphism of the common zinc transporter SLC30A8/ZnT8 may increase susceptibility to type 2 diabetes, providing novel insights into the role of zinc in diabetes [34].

The Zn cation is excreted at the basolateral side of enterocytes into the portal blood, where it is bound to albumin (60%), α-macroglobulin (30%) and transferrin (10%) [35], which distributes the metal in the body [36]. Of note, the expression of metallothioneins (MTs) depends on zinc levels in enterocytes. Among four known MT genes (MT-1–MT-4) [37], mainly MT-1 and MT-2 are expressed in the intestine [38], and combines zinc and copper in the intestine, and prevents their serosal surface transfer. Intestinal cells are sloughed with approximately a 6-day turnover, and the metallothionein-bound copper and zinc are lost in the stool and are thus not absorbed [39]. Detailed processes of cellular distribution of zinc into enterocytes and its transfer through the cells after its absorption are not yet completely understood [2].

Plasma or serum zinc levels in healthy individuals ranges from 12 to 16 μM [40], and corresponds to 1% (or less) of whole-body zinc. Serum contains only 0.1% of the whole-body zinc, but the circulating zinc turns over rapidly to meet tissue needs [10], thus serum is important in zinc homeostasis within the body. Conversely, zinc stored in skeletal muscle and bone has low turnover and availability [41]. The plasma and serum zinc are directed into the hepatic circulation, and then it is released into systemic circulation and deliver zinc into various tissues (Scheme 1).

Brain concentration of zinc is high, yet very little is known about the molecular mechanism of zinc and homeostasis in nervous system (Scheme 1). Zinc can use several pathways to enter and/or exit brain. In neurons, zinc ions use (1) presynaptic release along with glutamate, (2) voltage-gated L-type Ca^2+^-channels and glutamate-gated channels and (3) a plasma membrane transporter mechanism [42].

The correct zinc balance is regulated by biliary and intestinal secretions, even if most zinc ions are reabsorbed. Additional routes of zinc excretion are through urine, feces and surface losses (sloughed skin, hair, sweat) [10].

## 3. Zinc Binding Proteins and Zinc Biological Role

The latest bioinformatic analysis showed that there are over 3000 proteins (~10% of all encoded proteins) that bind Zn ions [8], including enzymes, nuclear factors and hormones [43]. Zn is mainly bound to four amino acid residues and adopts a tetrahedral coordination, but the stability of the formed adducts depends on metal binding sites (most frequently sulfur from cysteine, nitrogen from histidine and oxygen from glutamate or aspartate) and surrounding protein and ligand environments. The affinity of zinc proteins for Zn ions and the stability of formed complexes determines Zn function, and how proteins regulate its mobility and cellular availability [44].

Zinc has a catalytic, structural and regulatory role in its adducts with proteins. According to Kochanczyk et al. [44] zinc proteins can be divided into five classes, based on correlation between structural and functional similarities of the metal binding center. In the first-class, the catalytic zinc binding domains coordinate metal with three amino acid (mainly histidine and aspartate/glutamate) donors deriving from a single polypeptide chain. The second-class proteins bind zinc in mononuclear, tetrahedral coordination sphere involving four protein-derived ligands (mainly cysteine–sulfur donors and histidine–nitrogen donors). The third-class proteins have multinuclear zinc binding sites, with more than one metal ion in distinct site. In the fourth-class proteins, ligand environment influences metal affinity and in the consequence the high mobility of zinc. The last class of zinc binding proteins coordinate metal intermolecularly with ligands present in two or more different polypeptide chains.

The zinc presence in different proteins lead to zinc involvement in numerous cellular processes and biochemical pathways including cytoprotection against reactive oxygen species and bacterial toxins, regulation of multiple transcription factors, restore of dermal and mucosal barrier integrity, and production of antibodies and circulating lymphocytes. Immune function, wound healing, protein synthesis, DNA synthesis and cell division are the best-known processes where the zinc presence is essential. Taste and smell, growth and development during pregnancy, childhood, and adolescence are also controlled by the Zn homeostasis. Antioxidant and antibacterial properties of zinc protects against aging and accelerate wound healing [45]. Finally, different enzymes involved in epigenetic events, such as DNA methylation and histone modification, require zinc [46,47]. Therefore, zinc could be considered an essential trace metal in epigenetics events, which determine both health and disease conditions [48].

## 4. Zinc Pharmacodynamics and Clinical Trials

There are 69 registered clinical trials for the use of zinc therapy in different human pathologies [49] (Table S1—complete list of clinical trials; Table 1—selected clinical trials): 35 are completed, while others are going to recruit, or are already recruiting. Zinc therapy is proposed mostly for treatment, prevention or supportive care.

The use of oral zinc therapy showed effective results in boosting the immune system, treating the common cold and recurrent ear infections, as well as preventing lower respiratory tract infections [49]. Moreover, zinc can be used for the treatment and/or prevention of zinc deficiency and its consequences, for instance slowed wound healing, acute diarrhea in children, and stunted growth. Other indications for zinc oral therapy are (in alphabetic order): candidiasis; common cold; diaper dermatitis and rash; eye redness; iron deficiency; ocular and skin irritation; sunburn and Wilson’s disease [49].

Oral zinc therapy consists of zinc oxide or zinc inorganic salts, for instance zinc sulfate, zinc gluconate, zinc acetate and zinc picolinate. There is no significant difference in efficacy between different zinc salts, but it may affect patient tolerance. Most forms of zinc salts have nausea and epigastric distress as potential side effects. Moreover, it appears that some patients may not be zinc responsive, and adherence to therapy and careful monitoring are critical [50].

The safety of high-dose intravenous zinc (HDIVZn) has been presented in literature [51,52,53,54]. HDIVZn has been administered in the treatment of burns at doses ranging from 26.4 to 37.5 mg/d for 8 successive days without any side effects [51,52,53]. Oral zinc at doses over 75 mg/d has antiviral effects against common cold viruses, including influenza viruses [55]. Mild adverse effects of zinc supplementation have been reported with dosages above 200 mg/d [56,57].

The half-life of zinc in humans is approximately 280 days [58], while the clearance of zinc was found to be 0.63 ± 0.39 μg/min [59]. A pharmacokinetic study made on rats showed that zinc particles were mainly distributed to liver, lung and kidney within 72 h without significant gender differences [60]. According to the Toxnet database of the U.S. National Library of Medicine, the oral LD_50_ (measured in rats and mice) for zinc is close to 3 g/kg body weight [61].

Zinc can be chelated by ligands present in food products and metal binding sites of drugs (list of 50 potential drug interactions can be find in reference [49]). When assuming zinc, milk and phosphorous containing products should be avoided at least 2 h before administration. Moreover, zinc should not be assumed with bran and high fiber foods. For optimal absorption, zinc should be taken on an empty stomach, at least 1 h before and 2 h after eating. Zinc can be taken with food to reduce gastrointestinal upset [49].

Zn absorption can be reduced by malabsorptions, diarrheal episodes, pathogen translocations and celiac disease [62]. Of note, 10.4% patients with SARS-CoV-2 infection presented diarrhea [63].

## 5. Zinc in the Therapy of Wilson’s Disease

Wilson’s disease (WD) is an inherited metabolic disorder leading to hepatic and extrahepatic copper deposition. Most cases of WD occur in adolescents, but in some cases it can affect older people [64]. Diagnosis is usually established after the evaluation of serum ceruloplasmin (CP, which is typically below the reference range in WD), 24 h urinary Cu excretion and a slit lamp examination for the presence of Kayser–Fleischer rings (indicative of corneal Cu deposition). The histochemical demonstration of hepatic copper [65], genetic tests [66] and ultrastructural changes [67] are now important in the diagnosis of Wilson’s disease [68].

The liver synthesizes transporting P-type ATPases (ATP7B) protein and regulates copper metabolism. The WD copper concentration in hepatocytes varies during disease progression and reaches fivefold higher liver copper content (dry weight) in WD patients than that in healthy individuals [69]. The human brain has the highest concentration of copper after the liver [70] and copper concentration in the brain of WD patients is 10–15 fold higher than in healthy controls [71].

None of the drug treatments for WD can cure the disease, and prescribed treatments are life-long oral regimen. Each drug treatment aims at decreasing copper overload in the body, either by the copper chelation with D-penicillamine (DPen) [72] or trientine [73], or by zinc salts [74], which inhibit intestinal copper absorption through slow transcriptional induction of cellular metallothioneins [75]. After a lag phase, Zn also leads to excretion of copper from the body.

The first proposal to introduce oral zinc in the therapy of Wilson’s disease dates back to the early 1960s, and was contained in a thesis defended at the University of Amsterdam by the student G. Schouwink [76]. It was necessary to wait for 17 years for the first report in the international literature of the ability of zinc to decrease copper absorption in the gastrointestinal tract [77]. Sixty years after the first report, zinc is generally considered an alternative to chelating agents for the therapy of WD [78]. Monotherapy is generally considered one of the best options for young patients with WD around the world [79]. There are promising evidences that zinc therapy may decrease liver injury and provide antifibrotic effects in patients with WD [80].

In the recent study, Appenzeller-Herzog et al. [81] presented the comparative (prospective, retrospective, randomized and non-randomized) study of common therapies for WD, namely D-penicillamine (DPen), zinc salts, trientine and tetrathiomolybdate. It was shown that zinc therapy has similar effects to penicillamine in terms of prevention or reduction of hepatic or neurological WD symptoms, and at the same time is safer and has lower association with mortality. Severe side effects necessitating drug withdrawal were more frequent on DPen than on Zn [82]. Moreover, neurological deterioration after the copper-chelating therapy were more frequent when using DPen as compared to Zn [82]. Nevertheless, rare cases of gastrointestinal reactions and hepatic deterioration can occur during zinc therapy [83,84,85]. Up to now, eight patients with anemia after a long period of zinc therapy for WD have been reported [86], thus regular follow-up during zinc treatment and the involvement of specialists in the long-term management of Wilson’s disease are recommended.

According to the current guidelines [87], symptomatic patients should be treated with a chelating agent, although Zn may be used as first-line therapy in those with neurological disease [88,89]. In presymptomatic patients, either a chelator or Zn can be used [88,89]. Neurological deterioration is rare in zinc salts treatment [67], thus in patients with neurological symptoms worsening after treatment with chelators, zinc therapy should be introduced [90].

The recommended dose for adult patients is elemental zinc 150 mg/day in three divided doses, while dose recommended for children weighing less than 50 kg is 75 mg/day in three divided doses. Zinc salts should be assumed 1 h before or 2 h after meals for the optimal absorption and efficacy [87].

During gestation women should follow a low-copper diet, and the therapy with copper chelating agents should be decreased to 70% of the normal dose [87]. There is no evidence that zinc is teratogenic, nevertheless zinc acetate therapy should be decreased to 75 mg/day in pregnancy [91]. Compared to copper chelating therapy, zinc lowers copper overload relatively slowly, thus avoids excessive copper removal and copper insufficiency during pregnancy [92]. The knowledge of zinc therapy during breastfeeding is scarce, but single case studies suggest good compliance [93].

The mechanisms by which zinc therapy ameliorates copper balance in WD patients are multiple and, in part, unknown. In the enterocytes, high Zn levels up-regulate transcription of metallothioneins through the induction of the metal-responsive transcription factor 1 (MTF1) [94]. The increase of MTs in the cytoplasm of enterocytes causes the sequester of absorbed copper, prevents transfer of copper atoms into the bloodstream, and thus decreases dietary copper absorption [75]. In this way, zinc can attenuate liver oxidative stress by introduction of metallothionein and inhibition of tumor necrosis factor (TNF). Moreover, Zn prevents decrease of glutathione (GSH) and glutathione peroxidase activity. Contemporary, zinc increases glutathione reductase activity in the liver and increased the expression of factors associated to hepatic apoptosis [50]. Other beneficial effect of zinc treatment in Wilson’s disease, and in general in all diseases characterized by copper toxicosis, have been recently described by Barber et al. [95].

## 6. Zinc in the Therapy of Non-Alcoholic Liver Disease (NAFLD)

Non-alcoholic fatty liver disease (NAFLD) is the most common chronic liver disease with 24.4% prevalence worldwide, with 20−30% dominance in Western countries, and 5−18% in Asian countries. NAFLD is a multifactorial disease with complex pathophysiology such as obesity (80% of obese individuals present NAFLD), insulin resistance (IR), dyslipidemia and metabolic syndrome [96,97]. Recently, it was shown that COVID-19 patients with NAFLD have increased risk of COVID-19 disease progression, underlined by an abnormal liver function, and by a longer period of viral shedding in comparison with non-NAFLD individuals [98].

Even if the pathogenesis of NAFLD remains uncertain, recent data demonstrated that zinc treatment might be preventive and/or protective factor in alcoholic as well as in NAFLD liver diseases. Liver maintains systemic zinc homeostasis in the body [99], thus chronic liver diseases can lead to zinc deficiency. Zinc deficiency may decrease free insulin-like growth factor-1, and increase iron overload in the liver, and therefore leads to chronic liver diseases via elevating lipid peroxidation [100,101].

For instance, non-alcoholic steatohepatitis (NASH) or cirrhosis in general, may alter the process of trace mineral metabolism, and decreased Zn level was associated with hepatic steatosis with a leptin receptor deficiency or dysregulation of a large number of genes in lipid metabolism [102,103]. Numerous studies demonstrated zinc deficiency in NAFLD patients [104,105], as well as in obese individuals and those with diabetes, and zinc could be involved in physiologic mechanism and disease progression. Recently, the relationship between zinc and hepatic fibrosis was revealed in patients with biopsy-proven NAFLD [106]. For these reasons, a decreasing trend of serum zinc level is a clinical tool to measure severity of NAFLD in terms of ultrasonographic aspect of hepatic steatosis and liver fibrosis [107,108].

Impaired liver hemostasis of zinc may lead to oxidative stress and inflammation of liver in NAFLD patients [109]. Zinc transporters play a significant role in attenuation of endoplasmic reticulum (ER)-stress and hepatic steatosis, which increases in zinc deficient patients [110]. In addition, zinc regulates secretion, receptor activation and signal transduction of insulin [111], thus zinc deficiency may influence insulin resistance and diabetes mellitus [112]. The recent studies by Fathi et al. [97] showed that 3-month therapy with 30 mg elemental zinc supplement improves serum levels of insulin, insulin resistance, superoxide dismutase 1 (SOD1) and malondialdehyde (MDA), thus improving stress status in overweight/obese NAFLD patients.

In this contest, the protective role exerted by zinc antioxidant enzymes in several liver diseases seems to be important also in NASH progression [113]. Moreover, Zn-treatment seems to have potential antiviral effect, restoring both innate and humoral immunity. Therefore, Zn treatment may also act in a synergistic manner, when co-administered with the standard antiviral therapy, in patients with hepatitis C, HIV, and SARS-CoV [114,115].

## 7. Zinc in the Prevention of COVID-19-Related Liver Injury

The severe stages of COVID-19 occur in up to 15% of patients, which develop acute respiratory distress syndrome (ARDS) or multiple organ failure (MOF) [116]. The SARS-CoV-2 infection may also develop hepatobiliary symptoms and enzyme elevation, which lead to liver injury. The cumulative prevalence of acute liver injury was calculated at 23.7 (16.1–33.1) per 100 patients with COVID-19 [117], with higher risk for males than females. Direct and/or indirect bilirubin, hepatocyte integrity markers (alanine aminotransferase (ALT) and aspartate aminotransferase (AST)), alkaline phosphatase (ALP), and gamma-glutamyltransferase (GGT) [118] and lactate dehydrogenase (LDH) [119,120] levels are reportedly higher in patients with COVID-19 [121,122], particularly in males. Moreover, younger age and elevated interleukin (IL)-6 or ferritin level have recently been defined as the strongest predictors of liver injury [123]. Of note, liver injury symptoms may appear even without respiratory symptoms, and persistent liver damage may advance over the patient’s entire lifetime [124].

Among the possible causes of liver injury are severe inflammatory response, anoxia, drug induced liver injury, direct cytotoxicity and pre-existing metabolic liver disease [125]. The activation of immune responses with the cytokine storm syndrome (increased T helper 12 (Th17) and CD8 T cells, Interleukin (IL)-2, IL-6, IL-7, IL-10, tumor necrosis factor-α, granulocyte-colony stimulating factor, interferon-inducible protein-10, monocyte chemotactic protein 1 and macrophage inflammatory protein 1 alpha) [116,126,127] [3,36,37]; together with dyshomeostastasis of hepatic biochemistry, hypoxia-reoxygenation (and hypovolemic shock due to severe dehydration), activation of Kupffer cells and oxidative stress, intestinal endotoxemia, sympathetic hyperactivity and adrenocortical system hyperactivity in patients with COVID-19 [128] contribute to liver injury. Moreover, ischemic/hypoxic liver injury is correlated to metabolic acidosis aggravation, calcium overloading and alterations in the mitochondrial permeability transition pore protein [129].

The angiotensin-converting enzyme 2 (ACE2) receptors is a key cell entrance for SARS-CoV-2, which directly contaminates hepatocytes and leads to moderate microvascular steatosis and mild hepatic lobular and portal activity [130,131,132]. ACE2 is a zinc metallopeptidase, with zinc ion at the catalytic site (1:1 metal/protein molar ratio) that bind and metabolize substrates. Low zinc concentrations (~10 μM; in vitro conditions) are indispensable for the proper enzyme functioning. Conversely, higher (~1 mM; in vitro conditions) zinc concentrations inhibit ACE2 activity [133].

The beneficial effects of zinc therapy against viral liver infections were ascertained in the treatment of *Hepatitis C* and *B* (HCV) and (HBV). Among different possible mechanism are (1) antioxidant properties of zinc, (2) balance between T helper 1 (TH1) and TH2 cells, (3) zinc enhancement of antiviral effects of interferon, (4) inhibitory effects of zinc in the HCV replicon system, and (5) hepatoprotective effects of metallothionein [134,135]. Moreover, Zinc therapy in HCV patients improves AST and ALT. Interestingly, patients with lower zinc concentrations showed later reduction in liver enzymes following zinc supplementation [50].

In coronaviruses, zinc inhibits both the proteolytic processing of replicase polyproteins and the RNA-dependent RNA polymerase (RdRp) activity [136,137,138,139]. The inhibition mechanism could be driven by zinc displacement of Mg^2+^ ions [140]; zinc binding to RdRp that induce a structural change in the conformation and disables RdRp to catalyze nucleotide incorporation; and/or high concentrations of zinc impairs viral polyprotein processing which is integral to virus replication [141]. Moreover, Zn acts as a membrane stabilizer that may directly prevent the entry of the virus into the cell [142].

High-dose intravenous zinc (HDIVZn) protects various organs, including the heart, kidneys and liver against the damage caused by hypoxia [143,144,145,146]. Zinc was shown to limit reactive oxygen species (ROS) through different mechanisms. Zinc induce MT mRNA and protein expression, which are involved in the intracellular defense against ROS and nitrogen species [147]. Moreover, zinc competes with Fe^2+^ and Cu^2+^ redox reactive ions for binding to cell membranes and proteins—and limits the production of hydroxyl radicals via Fenton chemistry. In addition, zinc upregulates the production and activation of antioxidant proteins, molecules and enzymes (e.g., glutathione, catalase and superoxide dismutase) [148]. Finally, zinc ions inhibit oxidant-promoting enzymes (e.g., nitric acid synthase and NADPH enzyme).

Elderly people are among the most vulnerable subjects, with higher risk for the development of severe form of Coronavirus disease-19 (COVID-19) [149]. The causes of this vulnerability remain, at the best of our knowledge, at least in part unknown. Among the multiple factors considered, old age-related weakening of the immune system, related to low zinc levels appears one of the most robust [150]. Zinc deficiency in the elderly was associated with decreased or diminished T-cell response, reduced natural killer cell activity, and depressed thymic hormone levels, which in turn increase risk of morbidity and mortality during respiratory infections [151]. Low zinc levels have been implicated in the pathogenesis of multiple diseases, including viral infections and gastrointestinal- and liver diseases [152]. Moreover, in vitro studies evidenced that high zinc levels may inhibit coronavirus replication [139]. Regarding infectious diseases, a zinc deficiency state is generally associated to a marked susceptibility to infections [153]. In clinical practice, serum copper-to-zinc ratio has been proposed as a tool for the evaluation of the health status. In particular, in older people, copper-to-zinc ratio should be considered a biomarker of the function of their immune system and their susceptibility to infections [154].

Zinc supplementation has been proposed for the management of patients affected by COVID-19 [155]. Accordingly, zinc supplementation might exert multiple health benefits to elderly people affected by COVID-19. The effects of zinc against SARS-CoV-2 infection, might be related to its ability to improve the immune response, minimize inflammation by protection against cytokine storm-induced multiorgan damage. Moreover, zinc supplementation inhibits viral replication and genome transcription, and decreases Angiotensin-Converting Enzyme 2 (ACE-2) expression through the downregulation of silent mating type information regulation 2 homolog (SIRT1) activity ending with a decrease in viral entry into the cells [156].

A recent study carried out on 3473 patients admitted to hospital with severe COVID-19, evidenced a relevant role in the administration of zinc with an ionophore in the outcome of patients. The 1006 patients who received Zn/ionophore therapy, zinc supplementation was associated with a 24% reduced risk of in-hospital mortality (12% of those who received Zn/ionophore died versus 17% who did not). Moreover, patients who received Zn/ionophore were discharged home with a higher incidence as compared to patients who did not receive the therapy [157]. Interestingly, in the same study, patients who received zinc alone or the ionophore alone did not show any significant improvement, suggesting that the association of zinc with ionophore have synergistic effects, and might represent a powerful tool for COVID-19 elderly patients.

The positive influence of zinc supplementation in patients with SARS-CoV-2 infection probably is not restricted to the virus itself. ACE, a zinc metalloproteinase, is also involved in the risk of several disease development [158]; therefore, this event may influence a possible Zn effect against COVID-19 infection. Zinc deficiency has been associated with an increased risk of developing atherosclerosis [159]; and enhancement of oxidative stress-related signaling processes in endothelial cells, causing endothelial dysfunction, and finishing with endothelial cell death. Experimental data in a mouse model of atherosclerosis, showed that low zinc in the diet promotes vascular inflammation and atherogenesis [160]. These data were at the basis of the recent hypothesis that COVID-19 might trigger the plaque vulnerability, transforming a stable plaque into a vulnerable one [161]. As a consequence, zinc supplementation in elderly COVID-19 patients, carriers of carotic or coronary atherosclerotic plaques, might halt the severe consequences due to the “activation” of the plaques caused by SARS-CoV-2 infection, improving significantly then prognosis in older patients. Zinc supplementation is recommended in all elderly patients involved in COVID-19 pandemic, in order to reinforce their immune competence and attenuate the dysfunctions caused, even at endothelial level, by the cytokine storm and by the multiple molecular pathways triggered by SARS-CoV-2 [161].

## 8. Conclusions

Zinc ion is bound by over 3000 proteins, and in this way it is involved directly or indirectly in many biochemical processes. Thus, zinc unbalance, mostly insufficiency, leads to numerous pathologies. Numerous clinical trials showed that zinc supplementation can be used not only in the prevention of diseases, but also as a treatment. Zinc pharmacological therapy is currently used in Wilson’s Diseases and showed positive results in the treatment of liver pathologies, namely non-alcoholic liver disease and COVID-19-related liver injury. Zinc is absorbed in the gastrointestinal tract and the proper diet is fundamental for zinc balance and health status.

## Figures and Tables

**Table 1:** Selected Zinc-based therapies in clinical trials (data adopted from https://clinicaltrials.gov/applying “Zinc therapy” to search results; accessed on 13 October 2021).

	NCT Number	Title	Status	Conditions	Interventions	Characteristics	Population	Locations
1	NCT04542993	Can SARS-CoV-2 Viral Load and COVID-19 Disease Severity be Reduced by Resveratrol-assisted Zinc Therapy	Active, not recruiting	•Covid19 •SARS-CoV Infection	•Dietary Supplement: Zinc Picolinate •Dietary Supplement: Resveratro •Dietary Supplement: Zinc Picolinate Placebo •Dietary Supplement: Resveratrol Placebo	Study TypeInterventional	Age: 18 Years to 75 Years (Adult, Older Adult)	•Swedish Medical Center, Seattle, Washington, United States
2	NCT04468139	The Study of Quadruple Therapy Zinc, Quercetin, Bromelain and Vitamin C on the Clinical Outcomes of Patients Infected With COVID-19	Recruiting	•Covid-19	•Drug: Quercetin •Dietary Supplement: bromelain•Drug: Zinc •Drug: Vitamin C	Study Type: Interventional	Age: 18 Years and older (Adult, Older Adult)	•Ministry of health. First health cluster, Riaydh, Riyadh, Saudi Arabia
3	NCT05003492	Utilizing the Crosstalk Among Aerosolized Phenformin, Methylene Blue, Photodynamic Therapy, Zinc and Potassium for Treating Severe COVID-19 Infection and Its Inflammatory Complication	Not yet recruiting	•COVID-19	•Combination Product: Combination therapy plus Standard therapy•Radiation: Photodynamic therapy •Drug: Standard therapy	Study Type: Interventional	Age: 18 Years to 70 Years (Adult, Older Adult)	•Ministry of health. First health cluster, Riaydh, Riyadh, Saudi Arabia
4	NCT04395768	International ALLIANCE Study of Therapies to Prevent Progression of COVID-19	Recruiting	•COVID19	•Dietary Supplement: Vitamin C •Drug: Hydroxychloroquine •Drug: Azithromycin. •Dietary Supplement: Zinc Citrate •Dietary Supplement: VitaminD3 •Dietary Supplement: Vitamin B12	Study Type: Interventional	Age: 18 Years and older (Adult, Older Adult)	•National Institute of Integrative Medicine, Melbourne, Victoria, Australia
5	NCT04447534	Zinc With Chloroquine/ Hydroxychloroquine in Treatment of COVID-19	Recruiting	•COVID	•Drug: Chloroquine •Drug: zinc	Study Type: Interventional	Age: 18 Years and older (Adult, Older Adult)	•Tanta university hospital, Assuit University, Ainshams University, Tanta, Egypt
6	NCT04828538	Vitamin D, Omega-3, and Combination Vitamins B, C and Zinc Supplementation for the Treatment and Prevention of COVID-19	Active, not recruiting	•Covid19	•Dietary Supplement: VitaminD •Dietary Supplement: Omega DHA/EPA •Dietary Supplement: Vitamin C, Vitamin B complex and Zinc Acetate	Study Type: Interventional	Age: 18 Years and older (Adult, Older Adult)	•Hospital de Soledad, San Luis Potosí, SLP, Mexico
7	NCT04370782	Hydroxychloroquine and Zinc With Either Azithromycin or Doxycycline for Treatment of COVID-19 in Outpatient Setting	Completed	•COVID-19	•Drug: Hydroxychloroquine •Drug: Azithromycin •Drug: Zinc Sulfate •Drug: Doxycycline	Study Type: Interventional	Age: 30 Years and older (Adult, Older Adult)	•St Francis Hospital, Roslyn, New York, United States
8	NCT04641195	Vitamin D and Zinc Supplementation for Improving Treatment Outcomes Among COVID-19 Patients in India	Recruiting	•COVID-19	•Dietary Supplement: Vitamin D3 (cholecalciferol) •Dietary Supplement: Zinc (zinc gluconate) •Dietary Supplement: Zinc (zinc gluconate) & Vitamin D(cholecalciferol) •Other: Placebo	Study Type: Interventional	Age: 18 Years and older (Adult, Older Adult)	•Saifee Hospital, Mumbai, Maharashtra, India •King Edward Memorial (KEM) Hospital, Pune, Maharashtra, India
9	NCT04621461	Placebo Controlled Trial to Evaluate Zinc for the Treatment of COVID-19 in the Outpatient Setting	Completed	•Corona Virus Infection	•Dietary Supplement: Zinc Sulfate 220 MG •Drug: Placebo	Study Type: Interventional	Age: 30 Years and older (Adult, Older Adult)	•St. Francis Hospital—The Heart Center, Roslyn, New York, United States
10	NCT00212368	Efficacy and Safety Study of Zinc Acetate to Treat Wilson’s Disease in Japan.	Completed	•Wilson’s Disease	•Drug: Zinc acetate	Study Type: Interventional	Age: 1 Year and older (Child, Adult, Older Adult)	
11	NCT00212355	Efficacy and Safety, Long-term Study of Zinc Acetate to Treat Wilson’s Disease in Japan.	Completed	•Wilson’s Disease	•Drug: NPC-02	Study Type: Interventional	Age: 1 Year and older (Child, Adult, Older Adult)	
12	NCT03659331	A Controlled Study of Potential Therapeutic Effect of Oral Zinc in Manifesting Carriers of Wilson’s Disease	Unknown status	•Wilson’s Disease	•Dietary Supplement: Zinc	Study Type: Interventional	Age: 18 Years and older (Adult, Older Adult)	
13	NCT02475928	Zinc Supplementation in Cirrhotic Patients	Unknown status	•Dysgeusia •Liver Cirrhosis	•Dietary Supplement: zinc gluconate •Dietary Supplement: Placebo •Behavioral: Nutritional education	Study Type: Interventional	Age: 18 Years to 70 Years (Adult, Older Adult)	•Medica Sur Clinic & Foundation, Mexico City, Mexico
14	NCT01899521	Examination of Zinc, S- adenosylmethionine, and Combination Therapy Versus Placebo in Alcoholics	Completed	•Alcoholism	•Procedure: Bronchoscopy •Dietary Supplement: Zinc sulfate 220 mg once daily •Dietary Supplement: Sadenosylmethionine 400 mg twice daily	Study Type: Interventional	Age: 18 Years to 60 Years (Adult)	•Atlanta VA Medical and Rehab Center, Decatur, GA, Decatur, Georgia, United States
15	NCT00149552	Zinc Therapy in HIV Infected Individuals Who Abuse Drugs	Completed	•HIV Infections •Substance-Related Disorders	•Dietary Supplement: zinc	Study Type: Interventional	Age: 18 Years and older (Adult, Older Adult)	•Camillus House, Miami, Florida, United States
16	NCT00449592	Oral Zinc Therapy for the Prevention of Mucositis	Completed	•Mucositis	•Drug: Zinc •Drug: Placebo	Study Type: Interventional	Age: 18 Years to 70 Years (Adult, Older Adult)	•Division of Hematology and Bone Marrow Transplantation, Sheba Medical Center, Ramat-Gan, Israel
17	NCT00325247	Efficacy of Zinc Therapy in Acute Diarrhoea in Young Children	Completed	•Acute Watery Diarrhea	•Drug: ZINC	Study Type: Interventional	Age: 1 Month to 59 Months (Child)	•ICDDR,B, Dhaka, Bangladesh
18	NCT01440608	Effectiveness of High-dose Zinc Therapy and Albendazole in the Treatment of Environmental Enteropathy	Completed	•Enteropathy	•Drug: Albendazole •Drug: Placebo •Dietary Supplement: High dose zinc	Study Type: Interventional	Age: 1 Year to 3 Years (Child)	•Saint Louis Nutrition Project, Blantyre, Malawi
19	NCT01162109	Zinc Therapy in Critical Illness	Active, not recruiting	•Severe Sepsis	•Dietary Supplement: Zinc sulfate	Study Type: Interventional	Age: 18 Years and older (Adult, Older Adult)	•University of Vermont College of Medicine, Burlington, Vermont, United States
20	NCT01259050	Safety Study of High Doses of Zinc in ALS Patients	Completed	•Amyotrophic Lateral Sclerosis	•Drug: Zinc and Copper	Study Type: Interventional	Age: 18 Years to 85 Years (Adult, Older Adult)	•Phoenix Neurological Associates, Phoenix, Arizona, United States
21	NCT00252304	Therapeutic Zinc in Childhood Pneumonia	Completed	•Pneumonia	•Drug: Zinc (zinc sulphate) •Drug: Placebo	Study Type: Interventional	Age: 2 Months to 35 Months (Child)	•Kanti Children Hospital, Kathmandu, Nepal
22	NCT00693680	Zinc Supplementation of Imipramine Therapy	Completed	•Major Depression	•Dietary Supplement: Zincas Forte •Dietary Supplement: Placebo	Study Type: Interventional	Age: 18 Years and older (Adult, Older Adult)	•Department of Psychiatry, Collegium Medicum, Jagiellonian University, Krakow, Poland
23	NCT02601742	Effectiveness of Oral Rehydration Therapy Supplemented With Zinc in the Management of Diarrhea Acute	Unknown status	•Diarrhea •Children	•Other: Zinc group •Other: Placebo group	Study Type: Interventional	Age: 6 Months to 5 Years (Child)	
24	NCT03923829	The Effect of Zinc on the Gingival Crevicular Fluid Level of Total Oxidant Capacity in Type 2 Diabetic Patients	Unknown status	•Chronic Periodontitis	•Drug: Zinc Sulfate •Procedure: scaling and root planing	Study Type: Interventional	Age: Child, Adult, Older Adult	

## Data Availability

Not applicable.

## Supplementary Materials

The following are available online at, Table S1: ClinicalTrials.gov Search Results 09/28/2021.
