# Supplementary material for: Zinc as a Drug for Wilson’s Disease, Non-Alcoholic Liver Disease and COVID-19-Related Liver Injury
Source: Molecules. 2021 Oct 31;26(21):6614. doi: 10.3390/molecules26216614 (PMC8587580; doi:10.3390/molecules26216614)
Supplement: Supplementary file 1 [file molecules-26-06614-s001.zip › molecules-1449699-supplementary.pdf]

ClinicalTrials.gov Search Results 09/28/2021

	NCT Number	Title	Status	Study Results	Conditions	Interventions	Characteristics	Population	Locations
1	NCT00149552	Zinc Therapy in HIV Infected Individuals Who Abuse Drugs	Completed	No Results Available	•HIV Infections<br>•Substance-Related Disorders	•Dietary Supplement: zinc	Study Type: Interventional	Age: 18 Years and older (Adult, Older Adult)<br>Sex: All	•Camillus House, Miami, Florida, United States
2	NCT00449592	Oral Zinc Therapy for the Prevention of Mucositis	Completed	No Results Available	•Mucositis	•Drug: Zinc<br>•Drug: Placebo	Study Type: Interventional	Age: 18 Years to 70 Years (Adult, Older Adult)<br>Sex: All	•Division of Hematology and Bone Marrow Transplantation, Sheba Medical Center, Ramat-Gan, Israel
3	NCT00325247	Efficacy of Zinc Therapy in Acute Diarrhoea in Young Children	Completed	No Results Available	•Acute Watery Diarrhoea	•Drug: ZINC	Study Type: Interventional	Age: 1 Month to 59 Months (Child)<br>Sex: All	•ICDDR,B, Dhaka, Bangladesh
4	NCT01440608	Effectiveness of High-dose Zinc Therapy and Albendazole in the Treatment of Environmental Enteropathy	Completed	No Results Available	•Enteropathy	•Drug: Albendazole<br>•Drug: Placebo<br>•Dietary Supplement: High-dose Zinc	Study Type: Interventional	Age: 1 Year to 3 Years (Child)<br>Sex: All	•Saint Louis Nutrition Project, Blantyre, Malawi
5	NCT01162109	Zinc Therapy in Critical Illness	Active, not recruiting	No Results Available	•Severe Sepsis	•Dietary Supplement: Zinc sulfate	Study Type: Interventional	Age: 18 Years and older (Adult, Older Adult)<br>Sex: All	•University of Vermont College of Medicine, Burlington, Vermont, United States
6	NCT04542993	Can SARS-CoV-2 Viral Load and COVID-19 Disease Severity be Reduced by Resveratrol-assisted Zinc Therapy	Active, not recruiting	No Results Available	•Covid19<br>•SARS-CoV Infection	•Dietary Supplement: Zinc Picolinate<br>•Dietary Supplement: Resveratrol<br>•Dietary Supplement: Zinc Picolinate Placebo<br>•Dietary Supplement: Resveratrol Placebo	Study Type: Interventional	Age: 18 Years to 75 Years (Adult, Older Adult)<br>Sex: All	•Swedish Medical Center, Seattle, Washington, United States
7	NCT01259050	Safety Study of High Doses of Zinc in ALS Patients	Completed	No Results Available	•Amyotrophic Lateral Sclerosis	•Drug: Zinc and Copper	Study Type: Interventional	Age: 18 Years to 85 Years (Adult, Older Adult)<br>Sex: All	•Phoenix Neurological Associates, Phoenix, Arizona, United States
8	NCT00252304	Therapeutic Zinc in Childhood Pneumonia	Completed	No Results Available	•Pneumonia	•Drug: Zinc (zinc sulphate)<br>•Drug: Placebo	Study Type: Interventional	Age: 2 Months to 35 Months (Child)<br>Sex: All	•Kanti Children Hospital, Kathmandu, Nepal

	NCT Number	Title	Status	Study Results	Conditions	Interventions	Characteristics	Population	Locations
9	NCT00693680	Zinc Supplementation of Imipramine Therapy	Completed	No Results Available	•Major Depression	•Dietary Supplement: Zincas Forte<br>•Dietary Supplement: Placebo	Study Type: Interventional	Age: 18 Years and older (Adult, Older Adult)<br>Sex: All	•Department of Psychiatry, Collegium Medicum, Jagiellonian University, Krakow, Poland
10	NCT02601742	Effectiveness of Oral Rehydration Therapy Supplemented With Zinc in the Management of Diarrhea Acute	Unknown status	No Results Available	•Diarrhea<br>•Children	•Other: Zinc group<br>•Other: Placebo group	Study Type: Interventional	Age: 6 Months to 5 Years (Child)<br>Sex: All	
11	NCT00530829	Impact of Home Zinc Treatment for Acute Diarrhea in Children	Completed	No Results Available	•Diarrhea	•Drug: zinc	Study Type: Interventional	Age: 2 Months to 4 Years (Child)<br>Sex: All	•Kenya Medical Research Institute, Kisumu, Kenya
12	NCT01210014	Systemic Zinc Sulphate in Treatment of Recurrent Aphthous Ulcerations:A Doubleblind, Placebo Controled Study	Completed	No Results Available	•Aphthous Stomatitis	•Drug: zinc sulphate 220mg/day in one dosage<br>•Drug: placebo: one dosage	Study Type: Interventional	Age: 10 Years and older (Child, Adult, Older Adult)<br>Sex: All	•Qazvin university of medical sciences, Qazvin, Iran, Islamic Republic of
13	NCT01468636	A RTC to Examine the Effectiveness of 400 mg of Oral Zinc Gluconate as Adjunctive Therapy for Ano-genital Warts	Terminated	No Results Available	•Genital Warts<br>•HPV	•Drug: Oral Zinc<br>•Drug: Placebo	Study Type: Interventional	Age: 19 Years and older (Adult, Older Adult)<br>Sex: All	•BC Centre for Disease Control, Vancouver, British Columbia, Canada
14	NCT00944359	Impact of Preventive and Therapeutic Zinc Supplementation Programs Among Young Children	Completed	No Results Available	•Zinc Deficiency<br>•Diarrhea<br>•Malaria	•Dietary Supplement: Daily preventive Zn; placebo treatment<br>•Dietary Supplement: Therapeutic Zn; daily placebo<br>•Dietary Supplement: Intermittent Zn; placebo treatment<br>•Other: Surveillance control group	Study Type: Interventional	Age: 6 Months to 27 Months (Child)<br>Sex: All	•Institut de Recherche en Science de la Sante, Bobo-Dioulasso, Burkina Faso
15	NCT00373100	The Efficacy of Zinc as Adjunct Therapy in the Treatment of Severe Pneumonia in Children	Completed	No Results Available	•Pneumonia	•Drug: Zinc acetate<br>•Drug: Placebo	Study Type: Interventional	Age: 6 Months to 59 Months (Child)<br>Sex: All	•Department of Paediatrics and Child Health, Makerere University, Kampala, Uganda
16	NCT00148733	CHIZAP: Community- and Health Facility-Based Intervention With Zinc as Adjuvant Therapy for Childhood Pneumonia	Completed	Has Results	•Pneumonia	•Drug: Zinc	Study Type: Interventional	Age: 2 Months to 3 Years (Child)<br>Sex: All	•Siddhi Memorial Hospital (SMH),Bhelukhel, Bhimsensthan, Bhaktapur, Nepal

	NCT Number	Title	Status	Study Results	Conditions	Interventions	Characteristics	Population	Locations
17	NCT00698386	Efficacy of Oral Zinc Administration as an Adjunct Therapy in New Pulmonary Tuberculosis (Category I) Patients	Unknown status	No Results Available	•Pulmonary Tuberculosis	•Dietary Supplement: Zinc supplement<br>•Dietary Supplement: Placebo	Study Type: Interventional	Age: 18 Years to 60 Years (Adult)<br>Sex: All	•All India Institute of Medcial Sciences-, New Delhi, India
18	NCT04468139	The Study of Quadruple Therapy Zinc, Quercetin, Bromelain and Vitamin C on the Clinical Outcomes of Patients Infected With COVID-19	Recruiting	No Results Available	•Covid-19	•Drug: Quercetin<br>•Dietary Supplement: bromelain<br>•Drug: Zinc<br>•Drug: Vitamin C	Study Type: Interventional	Age: 18 Years and older (Adult, Older Adult)<br>Sex: All	•Ministry of health.First health cluster ,Riaydh, Riyadh, Saudi Arabia
19	NCT05003492	Utilizing the Crosstalk Among Aerosolized Phenformin , Methylene Blue, Photodynamic Therapy , Zinc and Potassium for Treating Severe COVID-19 Infection and Its Inflammatory Complication	Not yet recruiting	No Results Available	•COVID-19	•Combination Product: Combination therapy plus Standard therapy<br>•Radiation: Photodynamic therapy<br>•Drug: Standard therapy	Study Type: Interventional	Age: 18 Years to 70 Years (Adult, Older Adult)<br>Sex: All	•Ministry of health.First health cluster ,Riaydh, Riyadh, Saudi Arabia
20	NCT00347386	Therapeutic Zinc in Infant Bacterial Illness	Completed	No Results Available	•Sepsis<br>•Bacterial Infections<br>•Pneumonia	•Drug: Drug: Zinc (zinc sulphate)<br>•Drug: Placebo	Study Type: Interventional	Age: up to 4 Months (Child)<br>Sex: All	•Deen Dayal Upadhyay Hospital,, New Delhi, India<br>•All India Institute Of Medical Sciences, New Delhi, India<br>•Kalawati Saran Children Hospital, New Delhi, India
21	NCT02044107	The Effectiveness of Co-packaging With Zinc to Improve Treatment of Diarrhea and Pneumonia in Guatemala	Unknown status	No Results Available	•Diarrhea.<br>•Pneumonia.	•Behavioral: Co-packaging and counseling messages	Study Type: Interventional	Age: 2 Months to 59 Months (Child)<br>Sex: All	•Guatemala: Ministry of Public Health and Social Assistance, San Marcos, Guatemala
22	NCT00198666	Efficacy of Zinc in the Treatment of Pneumonia	Completed	No Results Available	•Pneumonia	•Drug: Zinc	Study Type: Interventional	Age: up to 23 Months (Child)<br>Sex: All	
23	NCT04621461	Placebo Controlled Trial to Evaluate Zinc for the Treatment of COVID-19 in the Outpatient Setting	Completed	No Results Available	•Corona Virus Infection	•Dietary Supplement: Zinc Sulfate 220 MG<br>•Drug: Placebo	Study Type: Interventional	Age: 30 Years and older (Adult, Older Adult)<br>Sex: All	•St. Francis Hospital - The Heart Center, Roslyn, New York, United States
24	NCT03421314	Effect of Zinc and Selenium Supplementation on HIV+ Individuals on Antiretroviral Treatment.	Unknown status	No Results Available	•HIV<br>•ART<br>•Zinc Deficiency<br>•Selenium Deficiency<br>•Metabolic Complication<br>•Inflammation	•Dietary Supplement: Zinc gluconate and/or Selenium yeast	Study Type: Interventional	Age: 18 Years to 65 Years (Adult, Older Adult)<br>Sex: Male	•Centro de Investigaciones en Enfermedades Infecciosas, Mexico city, DF, Mexico

	NCT Number	Title	Status	Study Results	Conditions	Interventions	Characteristics	Population	Locations
25	NCT04641195	Vitamin D and Zinc Supplementation for Improving Treatment Outcomes Among COVID-19 Patients in India	Recruiting	No Results Available	•COVID-19	•Dietary Supplement: Vitamin D3 (cholecalciferol)<br>•Dietary Supplement: Zinc (zinc gluconate)<br>•Dietary Supplement: Zinc (zinc gluconate) & Vitamin D (cholecalciferol)<br>•Other: Placebo	Study Type:<br>Interventional	Age:<br>18 Years and older (Adult, Older Adult)<br>Sex:<br>All	•Saifee Hospital, Mumbai, Maharashtra, India<br>•King Edward Memorial (KEM) Hospital, Pune, Maharashtra, India
26	NCT01481181	An Efficacy Trial of a Gravity Fed Household Water Treatment Device as a Delivery System for Zinc	Completed	No Results Available	•Diarrhoea	•Dietary Supplement: Zinc enriched water	Study Type:<br>Interventional	Age:<br>2 Years to 45 Years (Child, Adult)<br>Sex:<br>All	•Maseno University, School of Public health, Kisumu, Western Kenya, Kenya
27	NCT02428647	Lao Zinc Study: Effects of Two Forms of Daily Preventive Zinc Versus Therapeutic Zinc Supplementation	Completed	No Results Available	•Diarrhea	•Dietary Supplement: MNP<br>•Dietary Supplement: preventive zinc supplement<br>•Dietary Supplement: therapeutic zinc supplement<br>•Dietary Supplement: preventive placebo supplement<br>•Dietary Supplement: therapeutic placebo supplement<br>•Dietary Supplement: placebo powder	Study Type:<br>Interventional	Age:<br>6 Months to 23 Months (Child)<br>Sex:<br>All	•National Institute of Public Health, Vientiane, Lao People's Democratic Republic
28	NCT01571856	Efficacy of Use of Zinc in the Treatment of Acute Diarrhea in Infants	Completed	No Results Available	•Acute Diarrhea<br>•Acute Gastroenteritis	•Drug: Zinc Sulfate<br>•Other: placebo	Study Type:<br>Interventional	Age:<br>1 Month to 6 Months (Child)<br>Sex:<br>All	•Centro Pediatrico Albina Patino, Cochabamba, Bolivia
29	NCT00513929	Zinc as Adjunct to Treatment of Pneumonia	Unknown status	No Results Available	•Pneumonia	•Dietary Supplement: Zinc sulphate	Study Type:<br>Interventional	Age:<br>2 Months to 59 Months (Child)<br>Sex:<br>All	•Baca Ortiz Children´s Hospitals, Quito, Ecuador
30	NCT00809809	Zinc for the Treatment of Herpes Simplex Labialis (HSL)	Completed	Has Results	•Herpes Simplex Labialis	•Drug: Zicam (Ionic zinc)<br>•Drug: placebo	Study Type:<br>Interventional	Age:<br>18 Years to 65 Years (Adult, Older Adult)<br>Sex:<br>All	•Steven Messer ND, DHANP, Tempe, Arizona, United States<br>•Deborah Thompson, MD MSPH, Santa Fe, New Mexico, United States<br>•Benjamin Kligler, MD, New York, New York, United States
31	NCT04370782	Hydroxychloroquine and Zinc With Either Azithromycin or Doxycycline for Treatment of COVID-19 in Outpatient Setting	Completed	No Results Available	•COVID-19	•Drug: Hydroxychloroquine<br>•Drug: Azithromycin<br>•Drug: Zinc Sulfate<br>•Drug: Doxycycline	Study Type:<br>Interventional	Age:<br>30 Years and older (Adult, Older Adult)<br>Sex:<br>All	•St Francis Hospital, Roslyn, New York, United States

	NCT Number	Title	Status	Study Results	Conditions	Interventions	Characteristics	Population	Locations
32	NCT02695160	Ascending Dose Study of Genome Editing by Zinc Finger Nuclease Therapeutic SB-FIX in Subjects With Severe Hemophilia B	Terminated	No Results Available	•Hemophilia B	•Biological: SB-FIX	Study Type: Interventional	Age: 18 Years and older (Adult, Older Adult)<br>Sex: Male	•Georgetown University Medical Center, Washington, District of Columbia, United States
33	NCT00682955	Comparing the Efficacy of Different Zinc Formulations in the Treatment of Diarrhea	Completed	No Results Available	•Incidence of Acute Diarrhea<br>•Incidence of Abdominal Pain	•Drug: Zinc Sulphate	Study Type: Interventional	Age: 6 Months to 5 Years (Child)<br>Sex: All	•Civil Hospital, Hyderabad, Sindh, Pakistan<br>•National Institute of Child Health, Karachi, Sindh, Pakistan
34	NCT01546805	Testing the Effect of Zinc Supplementation to Improve the Treatment Effect of Botulinum Toxin for Oculofacial Spasm	Unknown status	No Results Available	•Blepharospasm<br>•Hemifacial Spasm	•Drug: Zinc Supplement<br>•Drug: Sugar pill	Study Type: Interventional	Age: 18 Years and older (Adult, Older Adult)<br>Sex: All	•St Joseph's Hospital Hamilton, Hamilton, Ontario, Canada<br>•Carrot Eye Centre, Mississauga, Ontario, Canada
35	NCT04447534	Zinc With Chloroquine/ Hydroxychloroquine in Treatment of COVID-19	Recruiting	No Results Available	•COVID	•Drug: Chloroquine<br>•Drug: zinc	Study Type: Interventional	Age: 18 Years and older (Adult, Older Adult)<br>Sex: All	•Tanta university hospital, Assuit University, Ainshams University, Tanta, Egypt
36	NCT04828538	Vitamin D, Omega-3, and Combination Vitamins B, C and Zinc Supplementation for the Treatment and Prevention of COVID-19	Active, not recruiting	No Results Available	•Covid19	•Dietary Supplement: Vitamin D<br>•Dietary Supplement: Omega DHA / EPA<br>•Dietary Supplement: Vitamin C, Vitamin B complex and Zinc Acetate	Study Type: Interventional	Age: 18 Years and older (Adult, Older Adult)<br>Sex: All	•Hospital de Soledad, San Luis Potosí, SLP, Mexico
37	NCT00395226	Zinc Sulfate in the Treatment of Rosacea: A Randomized, Controlled Trial	Terminated	Has Results	•Rosacea	•Drug: zinc sulfate<br>•Drug: placebo	Study Type: Interventional	Age: 18 Years and older (Adult, Older Adult)<br>Sex: All	•St. Mary's Duluth Clinic Health System, Duluth, Minnesota, United States
38	NCT00325572	Evaluation and Treatment of Copper/Zinc Imbalance in Children With Autism	Terminated	Has Results	•Autism<br>•Pervasive Developmental Disorder	•Drug: oral zinc and vitamin C supplements<br>•Other: oral Placebo	Study Type: Interventional	Age: 3 Years to 8 Years (Child)<br>Sex: All	•Penn State Children's Hospital, Hershey, Pennsylvania, United States
39	NCT01783353	Oral Zinc Gluconate as Treatment for Recalcitrant Cutaneous Warts: A Randomized, Double-blind, Placebo-controlled Trial	Completed	No Results Available	•Recalcitrant Cutaneous Warts	•Dietary Supplement: Zinc gluconate<br>•Dietary Supplement: Corn starch pill	Study Type: Interventional	Age: 19 Years and older (Adult, Older Adult)<br>Sex: All	•Philippine General Hospital, Manila, NCR, Philippines

	NCT Number	Title	Status	Study Results	Conditions	Interventions	Characteristics	Population	Locations
40	NCT03659331	A Controlled Study of Potential Therapeutic Effect of Oral Zinc in Manifesting Carriers of Wilson Disease	Unknown status	No Results Available	•Wilson Disease	•Dietary Supplement: Zinc	Study Type: Interventional	Age: 18 Years and older (Adult, Older Adult)<br>Sex: All	
41	NCT01140074	Efficacy of Zinc Sulfate With Probiotics for the Treatment of Acute Diarrhea in Children	Unknown status	No Results Available	•Acute Watery Diarrhoea	•Drug: Zinc Sulfate	Study Type: Interventional	Age: 1 Month to 36 Months (Child)<br>Sex: All	•Szpital im Sw Jadwigi w Trzebicy, Trzebnica, Poland<br>•Klinika Pediatrii i Chorob Infekcyjnych Akademii Medycznej we Wroclawiu, Wroclaw, Poland
42	NCT03158168	Intralesional Candidal Antigen Versus Intralesional Zinc Sulphate in Treatment of Cutaneous Warts	Unknown status	No Results Available	•Warts	•Drug: Candida Antigen<br>•Drug: Zinc Sulfate	Study Type: Interventional	Age: 5 Years to 50 Years (Child, Adult)<br>Sex: All	•Assiut University Hospital, Assiut, Egypt
43	NCT04801433	Evaluate the Efficacy and Safety of Boroda Supramolecular Active Zinc in the Treatment of Scalp Psoriasis	Completed	No Results Available	•Scalp Psoriasis	•Other: Boleda Supramolecular Active Zinc Conditioner<br>•Drug: Capotetriol scalp solution<br>•Other: Supramolecular Hydrogel	Study Type: Interventional	Age: 18 Years to 65 Years (Adult, Older Adult)<br>Sex: All	•Dermatology Derpartment of Xijing Hospital, Xi'an, Shaanxi, China
44	NCT01662089	The Efficacy in Treatment of Female Pattern Hair Loss Using 5% Minoxidil Solution Combinded With Zinc Supplement	Unknown status	No Results Available	•Female Pattern Alopecia	•Drug: 15 mg Chelate zinc supplement<br>•Drug: Placebo drug supplement	Study Type: Interventional	Age: 18 Years and older (Adult, Older Adult)<br>Sex: Female	•Siriraj Hospital, Bangkok, Thailand
45	NCT04335877	Effect of Prompting the Supply of Zinc/LO-ORS Co-packs in the Private Sector Plus BCI on Childhood Diarrhea Treatment	Not yet recruiting	No Results Available	•Diarrhea, Infantile	•Other: Private sector component + modified BCC	Study Type: Interventional	Age: 2 Months to 60 Months (Child)<br>Sex: All	

	NCT Number	Title	Status	Study Results	Conditions	Interventions	Characteristics	Population	Locations
46	NCT01044654	Phase 1 Dose Escalation Study of Autologous T-cells Genetically Modified at the CCR5 Gene by Zinc Finger Nucleases in HIV-Infected Patients	Completed	No Results Available	•HIV Infection<br>•HIV Infections	•Genetic: SB-728-T	Study Type: Interventional	Age: 18 Years and older (Adult, Older Adult)<br>Sex: All	•UCLA Center for AIDS Research and Education, Los Angeles, California, United States<br>•Orange Coast Medical Group, Newport Beach, California, United States<br>•Quest Clinical Research, San Francisco, California, United States<br>•Circle CARE Center, LLC, Norwalk, Connecticut, United States<br>•Orlando Immunology Center, Orlando, Florida, United States<br>•Central West Clinical Research, Inc., St Louis, Missouri, United States<br>•Southwest CARE Center, Santa Fe, New Mexico, United States<br>•Ricky K Hsu, MD, PC, New York, New York, United States<br>•Gordon Crofoot, MD, PA, Houston, Texas, United States
47	NCT04654091	Cryotherapy VS. Nitric-zinc Complex in the Treatment of Plantar Warts.	Not yet recruiting	No Results Available	•Plantar Wart	•Procedure: Cryoteraphy with liquid nitrogen<br>•Procedure: Nitric-zinc complex	Study Type: Interventional	Age: 12 Years and older (Child, Adult, Older Adult)<br>Sex: All	
48	NCT02800369	Study of Molecular-targeted Therapy Using Zinc Finger Nuclease in Cervical Precancerous Lesions	Unknown status	No Results Available	•Human Papillomavirus-Related Malignant Neoplasm	•Biological: ZFN-603 and ZFN-758	Study Type: Interventional	Age: 18 Years to 50 Years (Adult)<br>Sex: Female	•Tongji Hospital, Wuhan, Hubei, China
49	NCT01899521	Examination of Zinc, S-adenosylmethionine, and Combination Therapy Versus Placebo in Alcoholics	Completed	No Results Available	•Alcoholism	•Procedure: Bronchoscopy<br>•Dietary Supplement: Zinc sulfate 220 mg once daily<br>•Dietary Supplement: S-adenosylmethionine 400 mg twice daily	Study Type: Interventional	Age: 18 Years to 60 Years (Adult)<br>Sex: All	•Atlanta VA Medical and Rehab Center, Decatur, GA, Decatur, Georgia, United States
50	NCT04395768	International ALLIANCE Study of Therapies to Prevent Progression of COVID-19	Recruiting	No Results Available	•COVID19	•Dietary Supplement: Vitamin C<br>•Drug: Hydroxychloroquine<br>•Drug: Azithromycin<br>•Dietary Supplement: Zinc Citrate<br>•Dietary Supplement: Vitamin D3<br>•Dietary Supplement: Vitamin B12	Study Type: Interventional	Age: 18 Years and older (Adult, Older Adult)<br>Sex: All	•National Institute of Integrative Medicine, Melbourne, Victoria, Australia

	NCT Number	Title	Status	Study Results	Conditions	Interventions	Characteristics	Population	Locations
51	NCT03923829	The Effect of Zinc on the Gingival Crevicular Fluid Level of Total Oxidant Capacity in Type 2 Diabetic Patients	Unknown status	No Results Available	•Chronic Periodontitis	•Drug: Zinc Sulfate<br>•Procedure: scaling and root planing	Study Type: Interventional	Age: Child, Adult, Older Adult<br>Sex: All	
52	NCT00036881	Zinc Sulfate in Preventing Loss of Sense of Taste in Patients Undergoing Radiation Therapy for Head and Neck Cancer	Completed	No Results Available	•Dysgeusia<br>•Head and Neck Cancer<br>•Oral Complications<br>•Radiation Toxicity	•Dietary Supplement: zinc sulfate<br>•Other: placebo	Study Type: Interventional	Age: 18 Years and older (Adult, Older Adult)<br>Sex: All	
53	NCT03650036	Root Canal Treatment in Primary Molars With Necrotic Pulp Using Two Different Pulp Therapies	Active, not recruiting	No Results Available	•Dental Pulp Necrosis	•Drug: Pulp Therapy CTZ Paste<br>•Drug: Pulp Therapy ZOE Paste	Study Type: Interventional	Age: 3 Years to 9 Years (Child)<br>Sex: All	•Federal University of Piaui, Teresina, Piaui, Brazil
54	NCT00408356	Immunological and Clinical Responses to Zinc in Children With Diarrhoea	Completed	No Results Available	•Diarrhoea	•Drug: Zinc	Study Type: Interventional	Age: 6 Months to 24 Months (Child)<br>Sex: All	•ICDDR,B. Mirpur Field Site, Dhaka, Bangladesh
55	NCT01472211	Water-based Zinc Intervention Trial in Zinc Deficient Children	Terminated	No Results Available	•Zinc Deficiency<br>•Diarrhea	•Device: zinc enriching filter (LifeStrawFamily)<br>•Device: placebo filter<br>•Other: disinfection tablets	Study Type: Interventional	Age: 2 Years to 5 Years (Child)<br>Sex: All	•Hopital de Zone, Natitingou, Atacora, Benin
56	NCT00142285	Zinc Pneumonia Outpatient Trial in Children < 2 Years	Completed	No Results Available	•Pneumonia	•Drug: Zinc sulphate (20 mg)	Study Type: Interventional	Age: up to 23 Months (Child)<br>Sex: All	•Kamalapur Urban Site, ICDDR,B: Centre for Health & Population Research, Dhaka, Bangladesh
57	NCT01198587	Oral Zinc for the Treatment of Acute Diarrhea in US Children	Completed	Has Results	•Diarrhea<br>•Gastroenteritis	•Drug: Zinc Sulfate<br>•Drug: Placebo oral capsule	Study Type: Interventional	Age: 6 Months to 6 Years (Child)<br>Sex: All	•Children's Hospital Boston, Boston, Massachusetts, United States
58	NCT04278599	Effect of Oral Zinc Supplementation as an Adjuvant to Topical Corticosteroid in Oral Lichen Planus Patients	Recruiting	No Results Available	•Oral Lichen Planus	•Drug: Oral Zinc supplementation<br>•Drug: Oral placebo supplementation	Study Type: Interventional	Age: Child, Adult, Older Adult<br>Sex: All	•Post Graduate Institute of Dental Sciences, Rohtak, Haryana, India
59	NCT03366584	The Effect of #-Carotene, Vitamin D3 and Zinc on Hyaline Membrane Disease and Feeding Intolerance in Premature Neonates	Unknown status	No Results Available	•Hyaline Membrane Disease<br>•Necrotizing Enterocolitis of Newborn	•Dietary Supplement: Beta carotene<br>•Dietary Supplement: Vitamin D3<br>•Dietary Supplement: Zinc<br>•Drug: Dexamethasone	Study Type: Interventional	Age: 17 Years to 45 Years (Child, Adult)<br>Sex: Female	•Cipto Mangunkusumo General Hospital, Jakarta, DKI Jakarta, Indonesia

	NCT Number	Title	Status	Study Results	Conditions	Interventions	Characteristics	Population	Locations
60	NCT01025583	Oral Rehydration Solution With Zinc and Prebiotics in Acute Diarrhea	Completed	No Results Available	•Diarrhea	•Dietary Supplement: hypotonic oral rehydration solution<br>•Dietary Supplement: Hypotonic oral rehydration solution with Zn and prebiotics	Study Type: Interventional	Age: 3 Months to 36 Months (Child)<br>Sex: All	•Pediatric Office, Naples, Italy
61	NCT01553708	Effect of EGF With Silver Sulfadiazine Cream Compared With Silver Zinc Sulfadiazine Cream for Treatment of Burn Wound	Completed	Has Results	•Deep Partial Thickness Burn	•Drug: Epidermal growth factor with silver sulfadiazine cream<br>•Drug: Silver zinc sulfadiazine cream	Study Type: Interventional	Age: 18 Years to 60 Years (Adult)<br>Sex: All	•Burn Unit, Siriraj Hospital, Bangkok, Thailand
62	NCT01484067	Study of a Cold Sore Patch for the Treatment of Herpes Labialis	Completed	No Results Available	•Herpes Labialis	•Device: Patch (Compeed© Total CareTM Cold Sore Patch)	Study Type: Interventional	Age: 18 Years to 70 Years (Adult, Older Adult)<br>Sex: All	•Intertek CRS, Manchester, United Kingdom
63	NCT02475928	Zinc Supplementation in Cirrhotic Patients	Unknown status	No Results Available	•Dysgeusia<br>•Liver Cirrhosis	•Dietary Supplement: zinc gluconate<br>•Dietary Supplement: Placebo<br>•Behavioral: Nutritional education	Study Type: Interventional	Age: 18 Years to 70 Years (Adult, Older Adult)<br>Sex: All	•Medica Sur Clinic & Foundation, Mexico City, Mexico
64	NCT03404310	Zinc Sulfate for Human Papillomavirus (HPV)	Terminated	No Results Available	•Human Papilloma Virus	•Dietary Supplement: Zinc Sulfate<br>•Other: Placebo (Gelatin Tablet)	Study Type: Interventional	Age: 21 Years to 65 Years (Adult, Older Adult)<br>Sex: Female	•Spectrum Health, Grand Rapids, Michigan, United States
65	NCT00355043	Efficacy of Zinc in the Treatment of Bronchiolitis and Prevention of Wheezing Respiratory Illness in Children Less Than Two Years Old	Completed	No Results Available	•Bronchiolitis	•Drug: Zinc sulphate 20 mg	Study Type: Interventional	Age: 2 Months to 23 Months (Child)<br>Sex: All	•ICDDR,B, Dhaka, Bangladesh
66	NCT02433743	Impact of RUTF on Body Composition, Anemia and Zinc Status of PLWHA	Completed	No Results Available	•HIV-infection/Aids<br>•Malnutrition	•Dietary Supplement: Ready-to-use therapeutic food (RUTF)	Study Type: Interventional	Age: 18 Years to 78 Years (Adult, Older Adult)<br>Sex: All	•University Cheikh Anta Diop, Dakar, Senegal
67	NCT02101008	Disulfiram and Chelated Zinc for the Rx of Disseminated Mets Mel That Has Failed First Line Therapy	Completed	Has Results	•Melanoma	•Drug: disulfiram and chelated zinc	Study Type: Interventional	Age: 18 Years and older (Adult, Older Adult)<br>Sex: All	

	NCT Number	Title	Status	Study Results	Conditions	Interventions	Characteristics	Population	Locations
68	NCT00212368	Efficacy and Safety Study of Zinc Acetate to Treat Wilson's Disease in Japan.	Completed	No Results Available	•Wison's Disease	•Drug: Zinc acetate	Study Type: Interventional	Age: 1 Year and older (Child, Adult, Older Adult)<br>Sex: All	
69	NCT00212355	Efficacy and Safety, Long-term Study of Zinc Acetate to Treat Wilson's Disease in Japan.	Completed	Has Results	•Wilson's Disease	•Drug: NPC-02	Study Type: Interventional	Age: 1 Year and older (Child, Adult, Older Adult)<br>Sex: All	

U.S. National Library of Medicine | U.S. National Institutes of Health | U.S. Department of Health & Human Services
